# The Influence of Bone Marrow-Secreted IL-10 in a Mouse Model of Cerulein-Induced Pancreatic Fibrosis

**DOI:** 10.1155/2016/4601532

**Published:** 2016-05-23

**Authors:** Wey-Ran Lin, Siew-Na Lim, Tzung-Hai Yen, Malcolm R. Alison

**Affiliations:** ^1^Department of Gastroenterology and Hepatology, Chang Gung Memorial Hospital, Chang Gung University College of Medicine, Taoyuan 333, Taiwan; ^2^Department of Neurology, Chang Gung Memorial Hospital, Chang Gung University College of Medicine, Taoyuan 333, Taiwan; ^3^Department of Nephrology, Chang Gung Memorial Hospital, Chang Gung University College of Medicine, Taoyuan 333, Taiwan; ^4^Centre for Tumour Biology, Barts and The London School of Medicine and Dentistry, London EC1M 6BQ, UK

## Abstract

This study aimed to understand the role of IL-10 secreted from bone marrow (BM) in a mouse model of pancreatic fibrosis. The severity of cerulein-induced inflammation, fibrosis, and the frequency of BM-derived myofibroblasts were evaluated in the pancreas of mice receiving either a wild-type (WT) BM or an IL-10 knockout (KO) BM transplantation. The area of collagen deposition increased significantly in the 3 weeks after cerulein cessation in mice with an IL-10 KO BM transplant (13.7 ± 0.6% and 18.4 ± 1.1%,* p* < 0.05), but no further increase was seen in WT BM recipients over this time. The percentage of BM-derived myofibroblasts also increased in the pancreas of the IL-10 KO BM recipients after cessation of cerulein (6.7 ± 1.1% and 11.9 ± 1.3%,* p* < 0.05), while this figure fell in WT BM recipients after cerulein withdrawal. Furthermore, macrophages were more numerous in the IL-10 KO BM recipients than the WT BM recipients after cerulein cessation (23.2 ± 2.3 versus 15.3 ± 1.7 per HPF,* p* < 0.05). In conclusion, the degree of fibrosis, inflammatory cell infiltration, and the number of BM-derived myofibroblasts were significantly different between IL-10 KO BM and WT BM transplanted mice, highlighting a likely role of IL-10 in pancreatitis.

## 1. Introduction

Interleukin-10 (IL-10), an anti-inflammatory cytokine, is mainly expressed and secreted by a variety of cell types derived from bone marrow (BM) such as T cells, monocytes/macrophages, dendritic cells, B cells, and natural killer cells. The anti-inflammatory effect of IL-10 is through the inhibition of proinflammatory cytokines from monocytes/macrophages, which are involved in the recruitment of additional inflammatory cells to sites of injury or infection [1]. In the pancreas, IL-10 has also been shown to play an anti-inflammatory role. The pretreatment of IL-10 could decrease the severity of cerulein-induced acute pancreatitis in mice by inhibiting cellular necrosis [2]. This effect is not directly related to a decrease of pancreatic inflammation but most likely due to the inhibition of proinflammatory cytokines from local inflammatory cells. Moreover, the level of IL-10 in serum and* IL-10* mRNA expression in lung, liver, and pancreas have been found to increase during acute pancreatic inflammation. Blocking endogenous IL-10 production by pretreatment with an anti-IL-10 monoclonal antibody increased the severity of acute inflammation suggesting a protective role of endogenous IL-10 in acute pancreatitis [3]. In chronic pancreatitis, using IL-10 knockout (KO) mice, Demols et al. have demonstrated that IL-10 could modulate proliferation and fibrosis in a cerulein-induced chronic pancreatitis mouse model [4], while in a nonalcoholic fatty pancreas disease mouse model, IL-10 secreted from spleen can downregulate the severity of inflammation [5].

BM-derived cells have been shown to be involved in pathogenesis of human pancreatic diseases. In patients with pancreatic cancer, BM-derived stem cells traffic more extensively in the peripheral blood [6]. Moreover, these increased numbers of BM-derived cells are associated with raised levels of various interleukins, including IL-10 [7]. Among the various types of BM-derived cells, BM-derived myofibroblasts have been shown to participate in pancreatic fibrosis [8, 9]. However, whether BM-derived myofibroblasts are affected by endogenous cytokines of BM origin, that is, can BM-secreted cytokines influence the number of myofibroblasts derived from BM, is not clear. In an IL-10 KO inflammatory bowel disease mouse model, it has been demonstrated that wild-type (WT) BM transplantation can ameliorate the severity of inflammation in the intestine, suggesting that BM-secreted IL-10 plays an anti-inflammatory role [10]. Similarly, IL-10 KO mice have an increased degree of inflammation and fibrosis in chronic pancreatitis [4]; however, this study was performed on an IL-10 KO background, and the role of BM-secreted IL-10 in pancreatic fibrosis is not well explored. Whether BM-secreted IL-10 can attenuate inflammation and fibrosis in pancreatitis and, furthermore, can change the number of myofibroblasts derived from BM is still not known. The aim of this study was to examine the inflammation, fibrosis, and recruitment of BM-derived myofibroblasts in the context of an absence of IL-10 secreted by BM cells.

## 2. Material and Methods

### 2.1. Experimental Design

**Figure 1:**
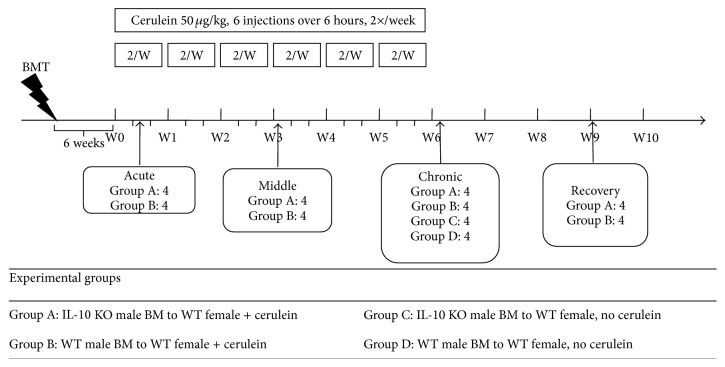
The study design for determining the role of bone marrow-secreted IL-10 in the cerulein-induced acute pancreatitis and pancreatic fibrosis mouse model. Bone marrow transplantation (BMT).

The procedures for animal experiments were performed under British Home Office procedural and ethical guidelines (Figure 1). Six-to-eight-week-old BALB/C WT female recipient mice (The Charles River Laboratory, Wilmington, Maine, USA) underwent whole body lethal gamma irradiation with 10 Gy in a divided dose four hours apart, to ablate their BM, followed immediately by tail vein injection of either WT (groups B and D) or IL-10 KO (groups A and C) male whole BM (2 × 10^6^ cells), resuspended in 0.1 mL sterile PBS with 2% FCS. After six weeks, the mice received either cerulein or vehicle (saline) injections to induce pancreatitis. There were four mice in each group. Acute pancreatitis was induced by 6 intraperitoneal injections of cerulein (50 *µ*g/kg) given at hourly intervals for 1 day. Pancreatic fibrosis was induced by the same protocol of cerulein injections being repeated twice a week for 3 and 6 consecutive weeks. Blood and tissues were harvested at 24 hours after the first injection on day 1 (acute groups), week 3 (middle groups), week 6 (chronic groups), and week 9 (recovery groups) for further analysis.

### 2.2. Scoring of Acute and Chronic Pancreatitis

The severity of acute pancreatitis was graded as previously reported by Van Laethem et al. [3]. The severity of chronic pancreatitis was graded as previously reported by Demols et al. [4]. The assessments were performed by two specialists independently in a blind manner.

### 2.3. Immunohistochemical Analyses

A mouse monoclonal anti-*α*-SMA antibody (A-2547, Sigma-Aldrich) and a mouse monoclonal anti-F4/80 antibody (MCA497, Serotec) were used to detect myofibroblasts and macrophages, respectively. Sections cut at 4 *µ*m were dewaxed, incubated in 1.8% v/v hydrogen peroxide in methanol for 15 min to block endogenous peroxidases, and then taken through graded alcohols to PBS. The sections were then incubated in 20% v/v acetic acid in methanol to block endogenous alkaline phosphatases.

Antigen retrieval treatment was performed by microwaving sections in BD Retrievagen A solution (550524, BD Pharmagen) for 10 minutes. To reduce nonspecific background staining, sections were next preincubated with 1% bovine serum albumin (A4503, Sigma-Aldrich) for 30 minutes. Primary antibodies were used at dilutions of 1 : 4000 for *α*-SMA and 1 : 100 for F4/80. The secondary antibody for *α*-SMA and F4/80 was a biotinylated polyclonal rabbit anti-mouse immunoglobulin used at a dilution of 1 : 300 (E0464, Dako). The tertiary layer was alkaline phosphatase-conjugated streptavidin (D0396; Dako) used at a 1 : 50 dilution. Slides were further developed in DAB and counterstained lightly with haematoxylin, dehydrated and mounted in DPX-type mount.

### 2.4. *In Situ* Hybridisation for Y Chromosome Detection

*In situ* hybridisation for Y chromosome-specific sequences in combination with immunostaining for *α*-SMA was performed to detect BM-derived myofibroblasts as previously reported [11] with slight modification. Briefly, sections were cut at 4 *µ*m and incubated in 1 M sodium thiocyanate (S7757; Sigma-Aldrich) to improve access of the probe to DNA. Following PBS washing, sections were digested in 0.4% w/v pepsin (P6887; Sigma-Aldrich) in 0.1 M hydrochloric acid to improve further access of the Y chromosome probe. The protease reaction was quenched in 0.2% w/v glycine (G4392; Sigma-Aldrich) in double concentration PBS and sections were then rinsed in PBS, postfixed in 4% w/v PFA (P6148; Sigma-Aldrich) in PBS, dehydrated through graded alcohols, and lastly air dried. A fluorescein isothiocyanate-labelled Y chromosome paint (1189-YMF-01; Cambio, Cambridge, UK) was used in the supplier's hybridisation mix. The probe mixture was added to the sections, sealed under glass with rubber cement, heated to 60°C for 10 minutes, and then incubated overnight at 37°C in a humidified chamber.

The next day, all slides were washed in 0.5x Standard Saline Citrate. The anti-FITC antibody (ab6656, Abcam) was applied in a dilution of 1 : 100 and slides further developed in DAB and counterstained lightly with haematoxylin, dehydrated and mounted in DPX-type mount.

### 2.5. Collagen Staining

Picrosirius red (VWR International) staining was used for intrapancreatic collagen detection. Quantitative analysis of collagen deposition was performed by digitized image analysis with NIH-Image J software [12]. The total pancreatic tissue area was distinguished from the background according to a difference in light density. The total amount of collagen (stained in red) was measured and expressed as a percentage of the total pancreatic surface.

### 2.6. Cell Counting

For each mouse pancreas, sections were analyzed by digitally photographing 10 consecutive microscope fields at ×400 total magnification. The numbers of myofibroblasts (*α*-SMA positive) and macrophages (F4/80 positive) were quantified and the proportion of BM-derived (Y chromosome positive) myofibroblasts was expressed as a percentage.

### 2.7. Statistics

Results are expressed as mean ± standard error of the mean (SEM). Mean values of the experimental groups were compared by Student's unpaired *t*-tests.* p* values of less than 0.05 were considered to be statistically significant.

## 3. Results

### 3.1. The Histological Changes in WT BM and IL-10 KO BM Transplanted Mice

**Figure 2:**
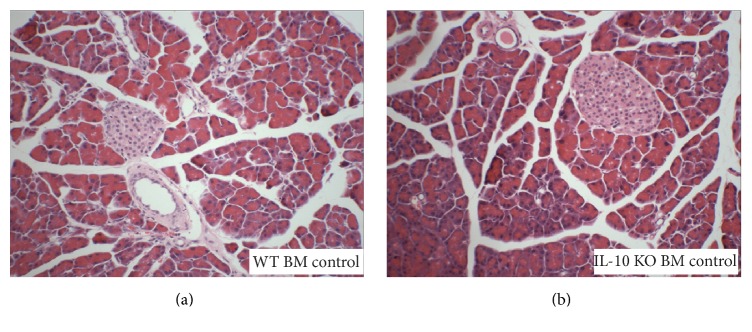
Histology of the pancreas from saline (control groups) treated female mice that received either wild-type (a) or IL-10 knock out (b) male BM transplants. H&E staining of the pancreas from the control group showed normal pancreatic structure.

In the saline-treated mice, the morphology of the pancreas was normal in mice receiving either WT BM or IL-10 KO BM (Figure 2). However, at 12 weeks after BMTx and 6-week saline injection, IHC staining with the F4/80 anti-macrophage antibody showed more infiltrated macrophages in the pancreas of mice receiving IL-10 KO BM (4.2 ± 0.3 per high power field (HPF, 400x)) compared to the mice receiving WT BM (1.9 ± 0.3 per HPF,* p* < 0.05) (Figures 7(a), 7(b), and 7(h)). This suggests that the lack of IL-10 secreted from the BM causes subtle inflammatory cell infiltration without any obvious histological changes.

**Figure 3:**
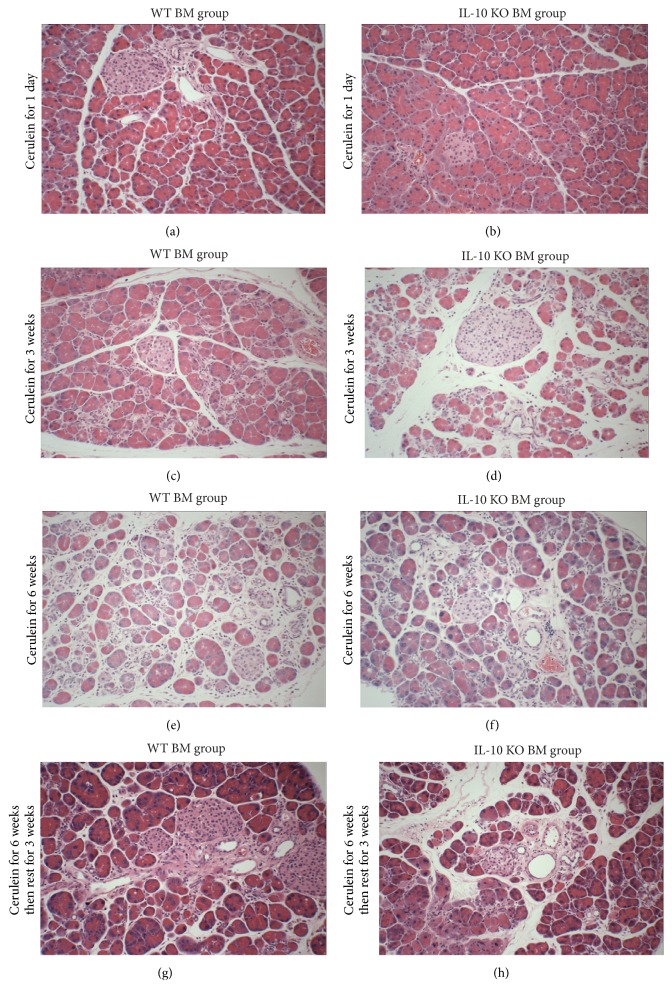
Histology of the pancreas from cerulein treated female mice that received either wild-type or IL-10 knocked out male bone marrow transplants. The development of mild acute pancreatic inflammation from 1-day cerulein treated mice ((a) and (b), 200x). Acinar atrophy, architectural changes, and pseudotubular complexes were found in mice receiving cerulein for 3 and 6 weeks and persisted for a further 3 weeks after cerulein injections ((c)–(h), 200x).

**Figure 4:**
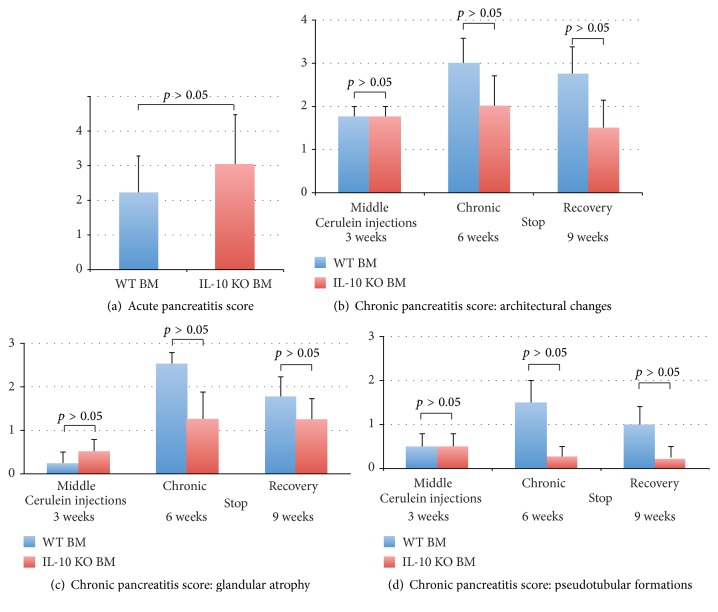
Histological scoring of acute pancreatitis (a) and chronic pancreatitis ((b)–(d)) in mice that received either a wild-type (blue bar) bone marrow or IL-10 knockout bone marrow (red bar) transplantation. Values represent the mean of scores ± standard error of mean (*n* = 4 mice per group).

After cerulein injection for 1 day, the morphology of the pancreas from the mice in both groups showed very mild focal oedema between lobules, inflammatory cell infiltration in the parenchyma but without collagen deposition, acinar atrophy, or structural change, which are the characteristic features of acute pancreatitis (Figures 3(a) and 3(b)). The acute pancreatitis histological score showed no significant differences between WT BM (2.3 ± 1.0) and IL-10 KO BM (3.0 ± 1.5,* p* > 0.05) groups (Figure 4(a)).

The atrophy of acinar cells, deformed architecture, pseudotubular complex formation, intralobular collagen deposition, and severe fibrotic change, which are the characteristic features of chronic pancreatitis were found not only in the pancreas of mice receiving 3 and 6 weeks of cerulein treatment, but also in the pancreas of mice that had 3 weeks rest after 6 weeks of cerulein (Figures 3(c)–3(h)). The histological scores of chronic pancreatitis including architectural changes, glandular atrophy, and pseudotubular complexes showed no significant differences between mice receiving WT BM and IL-10 KO BM, indicating that the lack of IL-10 secreted from the BM did not affect the severity of chronic pancreatitis, but there was a distinct trend for lower scores in the IL-10 KO BM administered mice (Figures 4(b)–4(d)).

### 3.2. Collagen Deposition in WT BM and IL-10 KO BM Transplanted Mice

One of characteristic features of chronic pancreatitis is collagen deposition. In the control groups, the percentage of collagen deposition (defined by Sirius red positive staining) to the total pancreatic area was 1.7 ± 0.2% and 1.8 ± 0.1% in mice that received a WT BM or an IL-10 KO BM transplant, respectively (*p* > 0.05). This result suggests that the lack of BM IL-10 does not affect normal collagen deposition.

**Figure 5:**
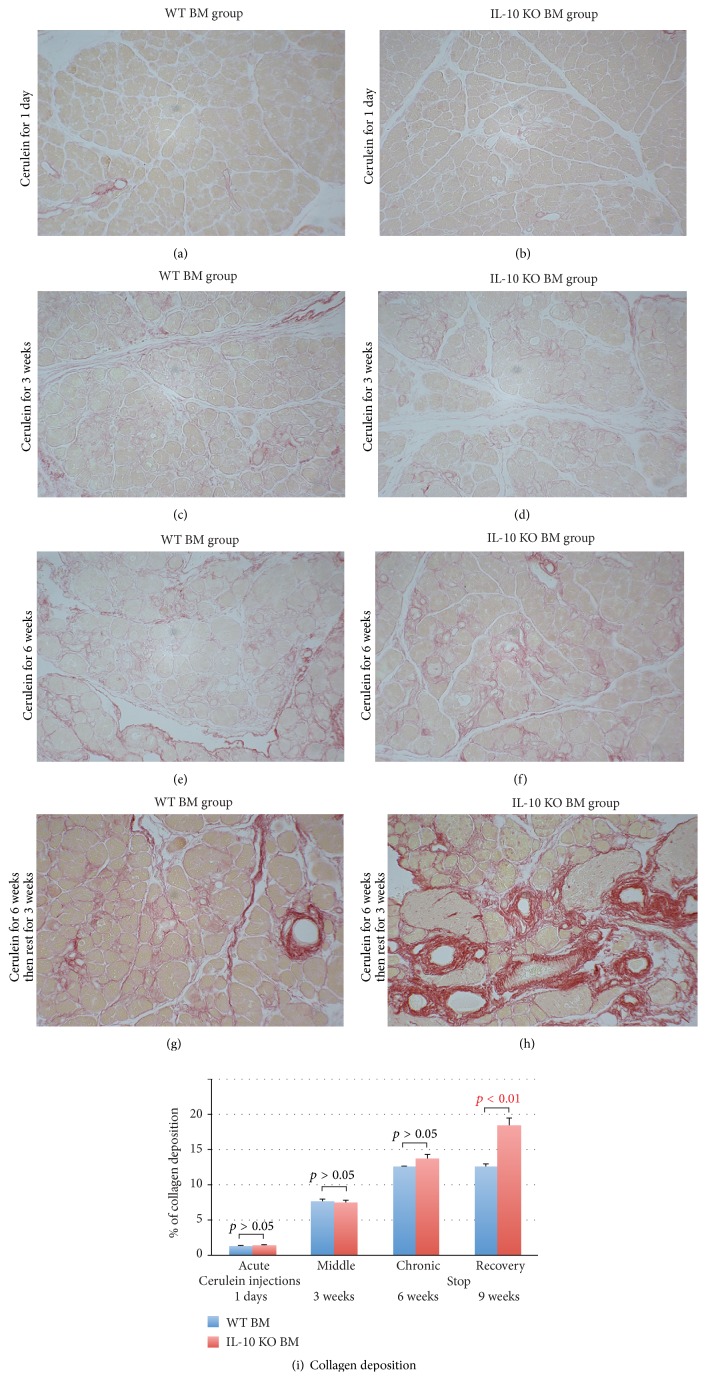
((a)–(h)) Sirius red staining of the pancreas from cerulein treated female mice that received either a wild-type male bone marrow or an IL-10 knock out male bone marrow transplant. (a) and (b) show normal collagen deposition between lobules after cerulein injection for 1 day. (c)–(h) show progressive collagen deposition with time. (i) Sirius red positive areas to the total area. Only in the recovery group is collagen deposition in the IL-10 knock out group significantly higher than that in the wild-type bone marrow group (*p* < 0.01).

Sirius red staining was also performed on all experimental groups (Figure 5). The 1-day cerulein injection did not cause further collagen deposition in either WT BM or IL-10 KO BM groups (Figures 5(a) and 5(b)), while continuous cerulein injections for 3 and 6 weeks induced progressive intralobular collagen deposition (Figures 5(c)–5(f)). Even following 3-week rest, the excessive intralobular collagen deposition remained (Figures 5(g) and 5(h)). In mice receiving a WT BM transplant, the areas occupied by collagen in the acute, middle, chronic, and recovery groups were 1.2 ± 0.2%, 7.6 ± 0.3%, 12.4 ± 0.2%, and 12.5 ± 0.4%, respectively. In mice receiving a IL-10 KO BM transplant, the areas were 1.4 ± 0.1%, 7.4 ± 0.4%, 13.7 ± 0.6%, and 18.4 ± 1.1%, respectively (Figure 5(i)). Similar degrees of collagen deposition were observed in mice with WT BM after 6 weeks of cerulein and following a further 3 weeks of recovery (12.4 ± 0.2% and 12.5 ± 0.4%,* p* > 0.05), suggesting that there was no immediate resolution of collagen deposition. On the other hand, the area occupied by collagen increased significantly in the 3 weeks after cerulein cessation in the mice with a IL-10 KO BM transplant (13.7 ± 0.6% and 18.4 ± 1.1%,* p* < 0.05), suggesting that the lack of IL-10 secreted from the BM results in further collagen deposition.

### 3.3. BM-Derived Myofibroblasts in the Pancreas of WT BM and IL-10 KO BM Transplanted Mice

**Figure 6:**
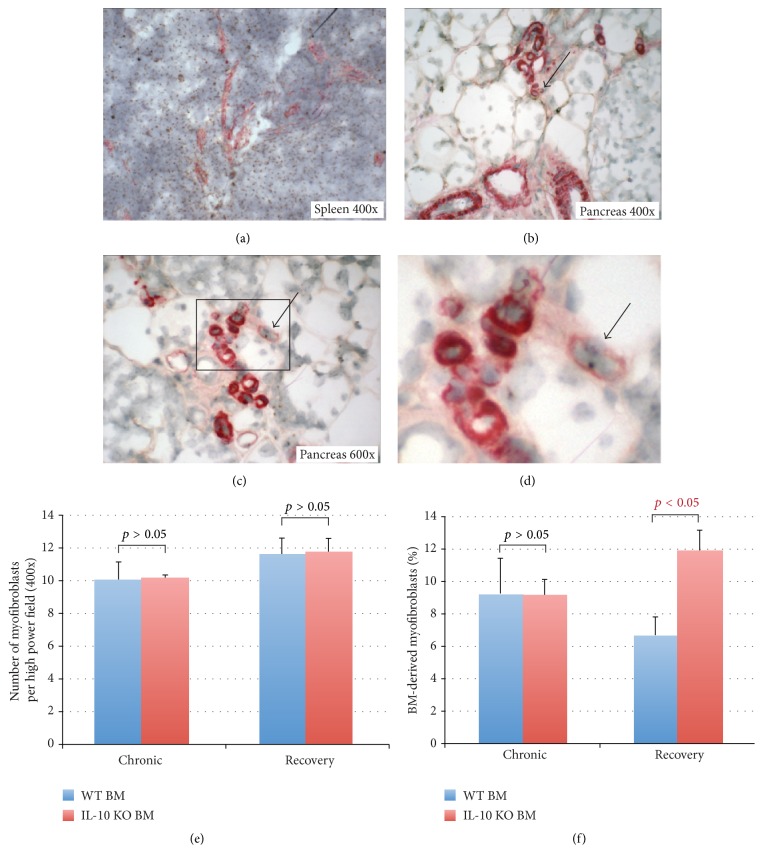
Bone marrow-derived *α*-SMA-positive cells in the pancreas from cerulein treated female mice that received either a wild-type (WT) or an IL-10 knock out (KO) male bone marrow (BM) transplant. (a) ISH for the Y chromosome in the spleen of female mice that received a WT male BM transplant. (b) IHC for *α*-SMA (red) and ISH for the Y chromosome shows BM-derived myofibroblasts in the pancreas from a mouse that received a WT male BM transplant (arrow, 400x) ((c) and (d)). Active myofibroblasts illustrated by *α*-SMA expression (red), while a small brown dot in the nucleus indicates this is a BM-derived myofibroblast in the pancreas from a mouse that received an IL-10 KO BM transplant (arrow). (e) The number of myofibroblasts per high power field (HPF) in mice receiving either a WT BM (blue bars) or IL-10 KO BM (red bars) in both the chronic and recovery groups. (f) The percentage of BM-derived myofibroblasts in the same groups.

Because increased collagen deposition was found in the IL-10 KO BM transplanted mice 3 weeks after the last cerulein injections, we wanted to know whether active myofibroblasts, the major collagen-producing cells in the pancreas, are also increased in number and are of BM derivation. Active myofibroblasts were identified by IHC for *α*-SMA in both WT BM and IL-10 KO BM transplanted mice in both the chronic and recovery groups. In order to detect BM-derived myofibroblasts, ISH for Y chromosome detection was then performed after the IHC for *α*-SMA (Figures 6(b)–6(d)). The spleen was used as a positive control of ISH for Y chromosome detection (Figure 6(a)), and the total number of myofibroblasts per high power field (HPF, 400x) was calculated. In mice receiving a WT BM transplant, the numbers of myofibroblasts per HPF in the chronic and recovery groups were 10.1 ± 1.1 and 11.6 ± 1.0, respectively. In mice receiving a IL-10 KO BM transplant, the numbers were 10.1 ± 0.2 and 11.7 ± 0.9, respectively (Figure 6(e)); thus, there were no differences between WT BM and IL-10 KO BM transplanted mice in the number of active myofibroblasts.

BM-derived myofibroblasts were readily recognised (Figures 6(b)–6(d)), and percentages were counted (Figure 6(f)). In mice receiving a WT BM transplant, the percentages of BM-derived myofibroblasts in the chronic and recovery groups were 9.3 ± 2.2% and 6.7 ± 1.1%, respectively. In the IL-10 KO BM recipients, the percentages were 9.2 ± 1.0% and 11.9 ± 1.3% (Figure 6(f)). Interestingly among the recovery groups, the percentage of BM-derived myofibroblasts was increased in the pancreas of the IL-10 KO BM recipients compared to those with WT BM (11.9 ± 1.3% versus 6.7 ± 1.1%,* p* < 0.05). This finding might suggest that BM-secreted IL-10 plays a role in suppressing BM cell transdifferentiation into myofibroblasts.

### 3.4. The Inflammatory Cell Response in the Pancreas of WT BM and IL-10 KO BM Transplanted Mice

IL-10 is considered as an anti-inflammatory cytokine; therefore it is reasonable to think that a lack of IL-10 secreted by the BM would result in a more severe pancreatitis. To investigate this, the numbers of macrophages were counted.

**Figure 7:**
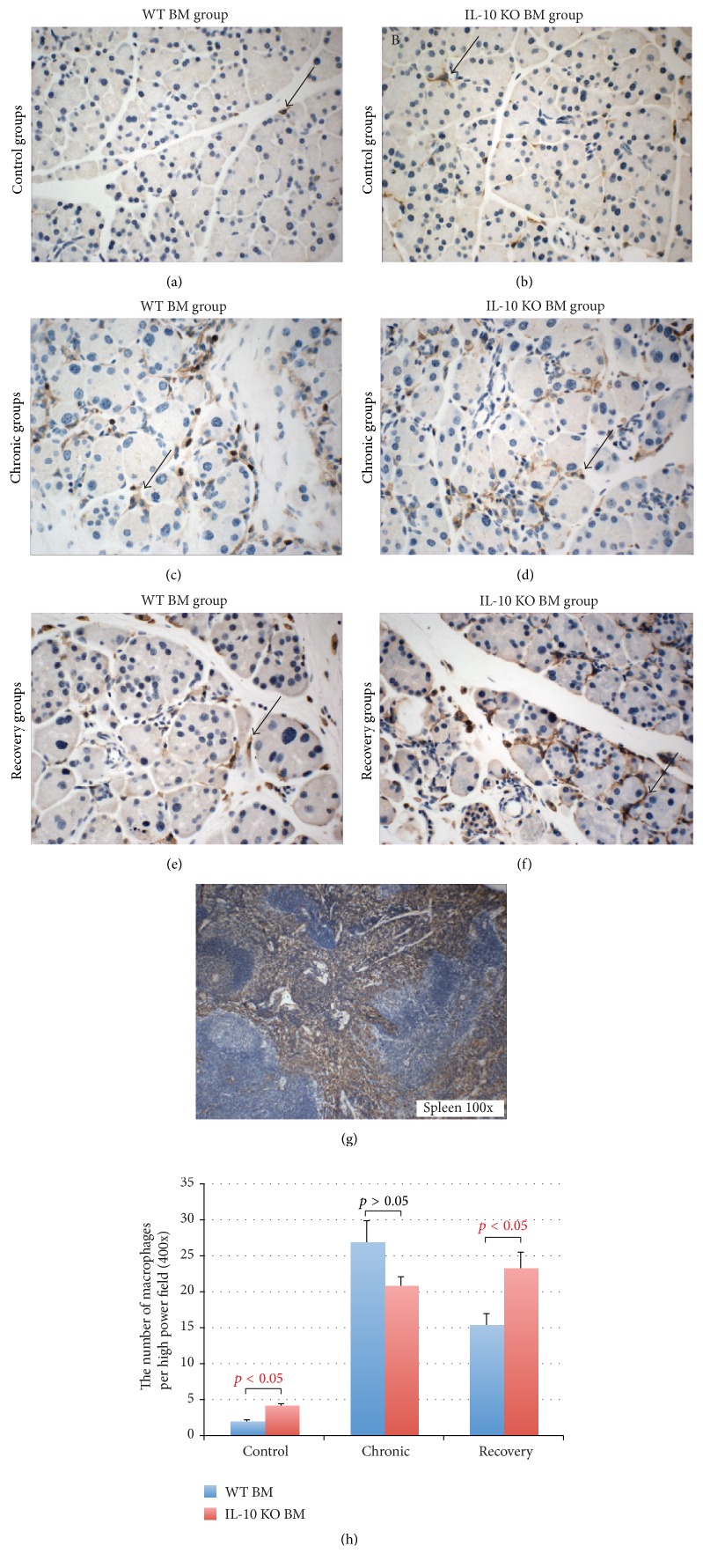
Immunohistochemical staining with F4/80 for macrophages in the pancreas from cerulein treated female mice that received either a wild-type (WT) male bone marrow (BM) or an IL-10 knock out (KO) male BM transplant. (a) and (b) show that in saline-treated mice (control groups), the F4/80 positive cells are rare in the pancreas of WT male BM transplanted mice (a), while the infiltration of F4/80-positive cells is increased in the pancreas of IL-10 KO male BM transplanted mice (b). (c) and (d) show that macrophage infiltration is significantly increased in the pancreas of both WT BM transplanted (c) and IL-10 KO BM (d) transplanted mice after receiving 6 weeks of cerulein injections (chronic groups). (e) and (f) show that macrophage infiltration was less in the WT BM transplanted mice after a 3-week recovery (e) but was unaltered in the IL-10 KO BM transplanted mice (f). (g) shows F4/80 staining in the spleen from a mouse that received a WT BM transplant but no cerulein. (h) shows the number of macrophages per HPF in mice receiving either a WT BM (blue bars) or an IL-10 KO BM (red bars).

Macrophages (F4/80-positive cells) are considered as chronic inflammatory cells contributing to tissue destruction, infiltrating the pancreas once acute pancreatitis is induced by cerulein. Staining with F4/80 in the spleen was used as a positive control (Figure 7(g)). Macrophages were very rare in the control pancreas of mice receiving WT male BM (1.9 ± 0.3 per HPF; Figures 7(a) and 7(h)), while in the controls with IL-10 KO male BM they were increased (4.2 ± 0.3 per HPF,* p* < 0.05; Figures 7(b) and 7(h)). In the mice with a WT BM transplant, the numbers of macrophages in the chronic and recovery groups were 26.8 ± 3.1 and 15.3 ± 1.7 per HPF, respectively (Figures 7(c), 7(e), and 7(h)), clearly a large decrease. In the mice with an IL-10 KO BM transplant, the number of macrophages in the chronic group was 20.8 ± 1.3 per HPF increasing to 23.2 ± 2.3 per HPF in the recovery group (Figures 7(d), 7(f), and 7(h)). Comparing the recovery groups, macrophage infiltration was significantly increased in the IL-10 KO BM recipients compared to the WT BM transplanted group (*p* < 0.05).

## 4. Discussion

The participation of BM-derived myofibroblasts has been well documented in pancreatic fibrosis using the experimental cerulein-induced pancreatitis mouse model [8, 9]. However, the influence of cytokines on these BM-derived fibrogenic cells has not been well clarified. Here we demonstrate for the first time that the lack of IL-10 secreted by BM cells is associated with progressive collagen deposition combined with increased numbers of BM-derived myofibroblasts and persistent infiltration of macrophages, all at 3 weeks after the cessation of cerulein treatment.

Pancreatic fibrosis is defined by the overgrowth and excess deposition of extracellular matrix components including collagen. It is caused by recurrent episodes of pancreatic parenchymal cell necrosis followed by fibrosis and is considered to be the end result of chronic pancreatitis induced by insults such as alcohol abuse, genetic disorders, pancreatic duct obstruction, recurrent acute pancreatitis, autoimmune pancreatitis, and unknown mechanisms [13]. Activated myofibroblasts serve as the primary collagen-producing cells and are believed to be the key cellular mediators of fibrosis in various organs [14]. Previous studies have also suggested that pancreatic myofibroblasts play an important role in the progression of pancreatic fibrosis [13, 15–18]. In the pancreas, multiple origins are considered as the source of activated myofibroblasts including pancreatic stellate cells [19], BM [8, 9], and fibrocytes [11]. Other studies have shown that BM-derived myofibroblasts contribute to the total population of activated myofibroblasts in the pancreas, with estimates varying from 6% to 20% in cerulein-induced chronic pancreatitis [8, 9]. Our experiments have also confirmed this finding since ~9% of myofibroblasts in the mice treated with cerulein for 6 weeks were derived from the BM. However, our results also showed that the percentage of BM-derived myofibroblasts increased in the IL-10 KO BM transplanted mice treated with cerulein for 6 weeks followed by a gap of 3 weeks, suggesting that IL-10 may play a role in the suppression of BM cells' transdifferentiation. Furthermore, the degree of fibrosis was also increased significantly in the IL-10 KO BM transplanted after the 3-week gap, suggesting that the increased number of BM-derived myofibroblasts may functionally contribute to pancreatic fibrosis. How IL-10 acts to suppress BM transdifferentiation into myofibroblasts is not answered in these experiments.

IL-10 has been shown to be a potent anti-inflammatory cytokine released during the course of experimental pancreatitis by decreasing the release of proinflammatory factors [2, 3, 20]. IL-10 levels were shown to be upregulated by Sonic hedgehog expression in the mouse model of cerulein-induced acute pancreatitis [21]. In this study, we further showed that a lack of IL-10 secreting BM cells could affect the inflammatory cell response during the recovery phase of cerulein-induced pancreatic fibrosis. The increased number of macrophages in the pancreas from the IL-10 KO BM transplanted mice is compatible with similar findings in skin wounds created on IL-10 KO mice [22].

Apart from an anti-inflammatory effect, IL-10 is also considered as an antifibrotic cytokine. It has been shown that the severity of fibrosis is increased in carbon tetrachloride-induced liver fibrosis in IL-10 KO mice [23]. Furthermore, the wound healing process is accelerated in IL-10 KO mice [22]. Increased numbers of *α*-SMA-positive myofibroblasts as well as macrophages were found in the skin wounds of IL-10 KO mice, suggesting that these two cell types enhance the contraction of wounds and mediate the accelerated tissue repair. In the cerulein-induced pancreatic fibrosis model, IL-10 KO mice have more severe histological lesions and fibrosis (intrapancreatic collagen content) than controls [4]. The plasma level of TGF-*β*1, a key stimulatory factor for myofibroblast activation, and the number of activated myofibroblasts were significantly higher in IL-10 KO mice, suggesting that the lack of IL-10 causes myofibroblast activation by increasing TGF-*β*1 that in turn increases the severity of fibrosis [4]. However, the results from this study were that the severity of fibrosis, the number of activated myofibroblasts, and inflammatory cells were the same in the IL-10 KO BM and WT BM transplanted mice after 6 weeks of cerulein. Differences were only revealed after a gap of 3 weeks from the last cerulein injection. A possible explanation is that IL-10 could be secreted from other cell types apart from BM cells during the episodes of acute pancreatitis induced by cerulein, and this is sufficient to maintain basic IL-10 levels during the acute inflammatory response. Indeed, although IL-10 is mainly secreted by BM-derived haematopoietic cells, it has been shown that IL-10 can also be detected in rodent hepatic stellate cells during the course of activation* in vitro* [24]. During the time without cerulein injection, the secretion of IL-10 from other cell types may decrease because of lack of inflammatory stimulation, and BM-secreted IL-10 may become predominant. The hypothesis that IL-10 secreted by other cell types can function well enough during acute inflammation is also supported by a previous study of IBD in IL-10 KO mice [10]; spontaneous colitis did not occur in WT mice that received the IL-10 KO BM transplantation, suggesting the IL-10 secreted from other cell types has a protective effect. Further experiments will be needed to clarify the significance of IL-10 secreted by other cell types.

## 5. Conclusions

The degree of fibrosis, the inflammatory cell response, and the number of BM-derived myofibroblasts were altered in the IL-10 KO BM transplanted mice in the recovery phase of pancreatic fibrosis after cerulein. These findings highlight a potential role of IL-10 on BM-derived myofibroblast behaviour and fibrosis in the pancreas.
